# Community Perspectives on Inequalities in the Provision of Basic Healthcare Services for the Most Vulnerable Populations in the Eastern Congo: A Qualitative Study

**DOI:** 10.1177/2752535X251321286

**Published:** 2025-02-17

**Authors:** Dieudonné Bwirire, Rik Crutzen, Rianne Letschert, Edmond Ntabe Namegabe, Bonfils Cheruga, Juliette Mukwege, Trésor Amisi Kasaya, Nanne de Vries

**Affiliations:** 1 Department of Health Promotion, CAPHRI Care and Public Health Research Institute, Faculty of Health, Medicine and Life Science, Maastricht University, Maastricht, The Netherlands; 2 Maastricht University, Maastricht, The Netherlands; 3 Faculté de Santé et Développement Communautaires, Université Libre des Pays des Grands Lacs, Goma, Democratic Republic of the Congo; 4 Research Initiatives for Social Development, Bukavu, Democratic Republic of the Congo

**Keywords:** basic healthcare service provision, universal health coverage, healthcare inequalities, community participation, community perspectives, democratic republic of Congo

## Abstract

**Background:**

There is a notable lack of evidence regarding the factors that shape the provision of essential healthcare services in post-conflict settings.

**Purpose:**

This study aimed to explore and describe the factors influencing the provision of basic health care services for the most vulnerable populations in the Eastern Congo.

**Method:**

Employing a qualitative research approach, twenty individual interviews with community members and thirteen focus group discussions were conducted. Participants were drawn from three geographically and demographically diverse locations with a history of decades-long armed conflicts in the Congo. Inductive thematic coding used the Health System Dynamics Framework categories (i.e. goals and outcomes, values and principles; service delivery; the population; the context; leadership & governance; and the organization of resources (finances; human resources; infrastructure and supplies; knowledge and information), while allowing for additional themes.

**Results:**

Our findings are presented thematically according to these ten categories. The following factors were perceived as key areas enabling or hindering healthcare provision to the community: (1) the context for organizing basic healthcare service delivery is complex and challenging; (2) the population plays a crucial role as an active producer of health and potential change agents; (3) there is a poor strategic policy framework to guide local-level communities in the provision of basic healthcare services; (4) several critical barriers and facilitators related to effective healthcare service delivery were identified; (5) the classification of basic health service delivery methods to meet the healthcare needs of the vulnerable population; (6) the healthcare system is pluralistic and consists of multiple overlapping systems and providers; and (7) service providers and potential service users still consider access to basic healthcare services challenging, potentially resulting in reduced coverage.

**Conclusion:**

These findings suggest that substantial changes in the factors contributing to the provision of basic healthcare services are necessary to ensure the delivery of basic healthcare services to the most vulnerable populations in the Eastern Congo. Consequently, there is a critical need to reconsider the healthcare delivery system, specifically addressing these contributing factors in the context of the Eastern Congo.

## Introduction

The past few years have witnessed a growing commitment by governments in low- and middle-income countries toward achieving Universal Health Coverage (UHC).^1,2^ UHC is a core principle of the United Nations (UN) Sustainable Development Goals (SDG 3)^3^ and covers essential health services, from health promotion and prevention to treatment, rehabilitation, and palliative care across the life course.^4^ UHC requires removing barriers to and improving the affordability, accessibility, and quality of health care and systems.^5^

There is a link between SDG3 and SDG10, especially concerning UHC. The health system influences the social determinants of health, enhancing health outcomes and tackling social inequalities.^6^ However, the link between UHC and health inequality reduction is not so clear-cut, as it has been shown that higher socioeconomic groups use more services and have greater access to health care.^7^

The transition from conflict to post-conflict complicates matters in terms of creating a stable political environment and shifting health service provision from a humanitarian to a developmental approach.^8^ Global public health challenges are concentrated in Fragile and Conflict-Affected Settings (FCAS), where health systems are constantly confronted by drivers of fragility, impacting the overall health system performance and leading to poor health outcomes.^9,10^ Hence, these systems have a limited capacity to respond to shocks.^11,12^ The provision of health services is commonly impeded by damaged health infrastructure and limited government stewardship, domestic financial resources, and health workforce, resulting in fragmented, uncoordinated, and inefficient health service delivery by a range of stakeholders with limited national coverage.^13,14^ Health services are often not equally distributed and are particularly weak for rural or scattered populations^15^ and there is a lack of minimum access to basic healthcare services for the most vulnerable groups.^16^

The Democratic Republic of Congo (DRC), for example, has been in a protracted state of fragility for the past three decades. The health system is fragmented and under-resourced, and disparities exist between urban and rural areas. For example, there is a concentration of doctors and nurses and medical facilities in urban areas; and these services are also better accessible. This is in contrast to rural areas where transportation methods are limited, both in terms of the availability of transport and the condition of roads, as well as issues related to security.^17^ There is a severe shortage of formal healthcare workers meaning that Community Health Workers play a key role in supporting the health system.^18^ More than 30 million Congolese do not have access to quality healthcare.^19^ In response to these challenges, the DRC formulated a comprehensive health system strengthening strategy (HSSS) in 2005 as a wide-ranging reform package under its strategy for growth and poverty reduction. The reforms, which are still being implemented, target health financing; partner and sector coordination; service delivery at the district level; public financing and government ownership of the sector strategy; the pharmaceutical sector; and the organic framework of the Ministry. These reforms are also allowing the defragmentation of services provided at the health district level, and are having an impact on all other levels of the health system.^20^

In establishing post-conflict health services, particular attention should be paid to providing services to individuals who have suffered most from conflict.^21^ Additionally, building in-country capacity in key policy and planning areas should begin as soon as possible after the conflict has ended,^22^ and equity should be seen as a core principle that should be developed to rebuild a health system that contributes effectively to reducing health inequalities. Importantly, a focus on equity means that high-quality healthcare needs to be available and affordable for all people, regardless of underlying social disadvantages.^23^ For this paper, *health inequalities* refer to socioeconomic differences in health that are avoidable, unnecessary, and unjust.^24^ For example, any measurable aspect of health that varies across individuals or according to socially relevant groupings can be called a health inequality.^25^ In contrast, *health inequities,* on the other hand, are a specific type of health inequality that denotes an unjust difference in health. For example, when health differences are preventable and unnecessary, allowing them to persist is unjust.^26^

An increasingly popular response to improve health service delivery in post-conflict countries is for the country’s government and international donors to jointly contract with non-governmental organizations to provide a Basic Package of Health Services for the country’s population. This approach is being applied in Afghanistan, Southern Sudan, and the DRC.^27,28^ Specifically for the DRC, a better understanding of factors influencing the provision of basic healthcare services (BHS) from the community perspective can inform policymakers and stakeholders of the successful implementation, and sustainability of basic healthcare delivery strategies.

This study aimed to explore and describe the factors influencing the provision of basic health care services for the most vulnerable populations in the Eastern Congo. To achieve this aim, the following research questions are addressed using the health system dynamics framework (HSDF): (1) How do people who have previously searched to access BHS experience inequality in health; (2) What are the factors that contribute to inequality in the provision of BHS, and (3) What are the past, current and future initiatives addressing health inequalities?

## Methods

### Study Setting

The DRC today is marked by persistent challenges that stem from its complex colonial history, ongoing political instability, and a legacy of poor governance. Since gaining independence from Belgium in 1960, the DRC has faced repeated conflicts, including civil wars and autocratic rule, which have deeply affected its efforts to establish a stable and democratic government. The impact of these conflicts continues to be felt, particularly in the eastern regions of the country, where violence, insecurity, and the presence of numerous armed groups persist. Thus, the eastern regions are among the poorest, most fragile, and insecure regions worldwide.^29^

The DRC’s wealth in natural resources, including diamonds, gold, coltan, vast hydropower potential, and extensive biodiversity, presents significant economic potential. However, this potential has been largely unrealized due to systemic corruption, poor governance, and the exploitation of resources by both domestic and foreign actors. Rather than driving development, these resources have often fueled conflict and exacerbated poverty, particularly in the eastern provinces of North and South Kivu.

Despite several key peace agreements, including those signed in 1999, 2002, and 2008, and subsequent efforts to build peace and stabilize the country, the DRC remains fragile. The ongoing violence, especially in the eastern regions, reflects a complex and militarized environment with multiple armed groups vying for control. The instability has led to a humanitarian crisis, with over 6.2 million displaced persons and a critical need for international aid. Many of these individuals live in extremely precarious conditions, facing multiple urgent needs, including access to food, clean water, sanitation, healthcare, and shelter.^30^ This suggests that eastern Congo is characterized by a complex blend of both conflict and post-conflict situations, often existing simultaneously rather than as distinct phases. The region’s situation is not dichotomous, but rather fluid, with periods of relative peace coexisting with ongoing instability.

This study was conducted in three different locations in the Eastern Congo, which were selected because they have experienced decades-long armed conflicts in the history of the DRC that have harmed the population’s health. The study locations included the Kivu provinces (South Kivu - a province with an estimated population of 7.3 million inhabitants), (North Kivu - a province with an estimated population of 10.6 million inhabitants), and Ituri (a province with an estimated population of 5.7 million inhabitants).^31^

### Inclusion and/or Exclusion Criteria

All community members were eligible unless they: (1) had not been living in Eastern Congo, or (2) had not experienced at least one episode of conflict in the last 10 years. A total of 20 community members were interviewed, 14 of whom were male and six female, with ages ranging from 28 to 51 years and an average participant age of 37 years. Additionally, 13 group discussions were organized with 139 community members, comprising 72 females and 67 males, with ages ranging from 16 to 65 years and above.

### Sampling and Recruitment Strategies

The study adopted a qualitative research approach^32^ to get a deeper understanding of the factors influencing the provision of basic healthcare services in the Eastern Congo, and to focus on the most relevant aspects of basic service delivery. It was also designed and reported according to the Consolidated Criteria for Reporting Qualitative Research Checklist (COREQ)^33^ (Additional File 1).

The participants in this study were community members, living in the Eastern Congo, and had experienced at least one episode of conflict in the last 10 years. A heterogeneous sample of community members was recruited, and study participants were selected purposively. Participants were recruited until data saturation.

We broadly define community members as individuals who belong to a specific entity or reside within a defined geographical area or boundary. These members typically share common health issues and receive services from the same healthcare providers. They are identified by characteristics such as shared interests and perspectives, a sense of place, joint actions, and social ties.^34^ Community members are well-positioned to identify evolving needs, anticipate forthcoming challenges, and motivate participation in health activities. They also have the potential to serve as leaders.

The Focus Group Discussions (FGDs) comprised members of the local community who were familiar with matters related to inequality in the provision of healthcare services. FGD participants consisted of men and women recruited from the local communities, including Internally Displaced People (IDP), elderly people, community liaison officers providers (referred to as the relais communautaire (RECO) in French), and other healthcare providers. Overall, 13 FGDs were conducted with community members of 16 years or older to explore their perceptions of what they have experienced as practice (s) / interventions used to address health inequalities in their respective communities.

To capture data in natural circumstances, participant observation was combined with FGDs. Notes from participant observation (field notes) were written directly into field notebooks.

To understand what is needed to address health inequalities among the most vulnerable populations in this environment, 20 individual interviews were conducted with community members who have more experience in this field. Participants were purposively sampled from different study settings and roles, and comprised community health managers and health center staff (such as physicians, pharmacists, nurses, and midwives). Using purposive selection for maximum between-group variation was planned with community members from the three study settings.

We used snowball sampling to identify participants from each group - the process starts with a small group of initial participants (seeds). These initial participants then refer the field researcher(s) to other potential participants they know within the target community. Those participants then refer the researchers to others, and so on. For this study, a convenience sample of “seeds” was first contacted by the community liaison officers (relais Communautaire) who solicited interest in participation. Eligible “seed” community members who consented to participate were informed about the study and asked to recruit others who met the study inclusion criteria. As such, eligible seeds agreed to select community participants, recruited eligible participants to act as referral agents for further participation, and recommended potential community participants for individual interviews and focus groups. Overall, 13 FGDs of approximately 8 to 12 male and female participants (for each FGD) were conducted with a total of 139 participants (67 male, 72 female).

### Data Collection

Data were collected using a semi-structured interview and a FGD topic guide (Additional Files 2 and 3). Three field researchers (BC, JM, and TAK) with qualitative research experience collected data between January to March 2021.

The researchers conducted one-on-one individual interviews with 20 participants to collect their perspectives on health inequalities in the provision of BHS to the most vulnerable population groups in these settings. Interviews were conducted in French or local languages depending on participant choice. Each interview lasted between 25 minutes to 1 hour and was audio-taped. All interviews were conducted by a trained local researcher (BC), who had many years of experience with the Congolese healthcare system. In preparation and support of the data analysis process, all interviews were digitally recorded using a voice recorder and transcribed verbatim by trained local research assistants (JM – in South and North Kivu, and TAK in Ituri) using Microsoft Word. Transcripts were finally reviewed for completeness and accuracy against interviews. Similar to the interviews, all FGDs were conducted in French or local languages depending on participant choice. They were also facilitated and recorded by BC, and transcribed verbatim by JM and TAK. In addition, field notes were taken by JM and TAK during each FGD to capture non-verbal communication.

### Data Analysis

With the consent of participants, data were collected during individual interviews (KIIs), group interviews (FGDs), and participant observations (field notes). Interviews and FGDs were analyzed inductively (i.e., themes generated from the data - open coding, creating categories, and abstraction) using Braun and Clarke’s phases of reflexive thematical analysis^35–37^ and Nvivo 12. Coding was done using Nvivo 12.

To allow the inclusion of any emerging issues in later interviews and support decisions on further data collection, data analysis (e.g. identifying, and reporting themes within data) started earlier and was ongoing throughout fieldwork.

All transcripts were then coded by at least two individuals (DB - the first author and PA- founder and methodology expert at the Center for Research Methods Consulting - an external consultant), which was later organized into similar codes to form themes and sub-themes. The codes were descriptions of key ideas identified while reading the transcripts. PA reviewed the codes that were developed, similar codes were clustered into different categories, and the categories were subsequently grouped into specific themes.

Trustworthiness was established during each of the six sequential phases^37^ of thematic analysis: (i) familiarizing ourselves with the data set by reading and re-reading the interview transcripts, (ii) generating initial codes after reading the transcript again, (iii) searching for themes, (iv) reviewing themes, (v) defining and naming themes, (vi) and producing the report (mainly supported by illustrative quotes).

### Rigour of This Study

We drew upon^38^ criteria of trustworthiness as a guiding tool for credibility, transferability, dependability, and confirmability to assess the rigour of this study:

i) *credibility* was ensured by comparing codes, based on the participants’ raw data that were used to generate themes and sub-themes. For instance, by prolonged engagement, observation, triangulation, and member check.^39^
ii) *transferability* was ensured by providing sufficient contextual information about the fieldwork settings and experience that would enable the reader to assess whether our findings are transferable to their setting.^39^
iii) *dependability* was achieved by ensuring that all the participants were asked the same questions,^39^ using a semi-structured interview guide (during KIIs) and FGD topic guide (Additional Files 2 and 3).
iv) *confirmability* was achieved by ensuring that the findings reported in this study emerged from the participants’ information and not the author’s imagination.^39^ This was assessed through all data collected from the interviews being audio-recorded and transcribed verbatim.

## Results

Several health systems frameworks have been published over the last decade. Many of the existing frameworks have a limited capacity to analyze the interactions and equilibriums between different elements of a health system. Given that there is no single set of best practices that can be put forward as a health system-strengthening framework meeting the highest standards or applicable to a post-conflict setting, we adopted the health system dynamics framework (HSDF)^40^ to describe community perspectives on inequalities in the provision of basic healthcare services for the most vulnerable populations in the Eastern Congo. Although this framework has its limits in meeting the highest standards or applicability to a post-conflict setting, it goes further than most frameworks and therefore it is helpful and supports our research questions, particularly because (i) it incorporates elements of existing frameworks, such as WHO building blocks,^41^ which have been considered as (i) the gold standard in this field, and (ii) it consists of 10 elements and their dynamic interactions: (1) goals and outcomes; (2) values and principles; (3) service delivery; (4) the population; (5) the context; (6) leadership & governance; and 7-10) the organization of resources (finances; human resources; infrastructure and supplies; knowledge and information). Our findings are reported narratively and structured according to these 10 elements of the HSDF.

### Socio-Demographic Attributes of Included Participants

In this study, we performed 20 key interviews and 13 FGDs with different community members living in the Eastern Congo. Interview participants were mostly male (14 male vs Six female), and ranged in age from 28 to 51 years, with an average participant age of 37 years. More than half of the FGD participants were female (72 female vs 67 male), and ranged in age from 16 to 65 years (and above). Detailed descriptions of the participants’ category by study setting are provided in Table 1 (Insert Table 1 here).

**Table 1.** Participants Category by Study Setting in Eastern Congo.

Eastern Congo provinces	Study site	Interviews key informants	FGDs with community members
North kivu	Location		
	*Goma (Mapendo)*	**1**	**1**
	*Goma (Jeunes)*	**1**	**1**
	*Mushake (Masisi)*	**1**	**1**
	*Bujovu (Nyiragongo)*	**2**	**1**
	*Shasha (Kirotche)*	**1**	**1**
South kivu	Location		
	*Bukavu 1 (Nkafu)*	**2**	**1**
	*Bukavu 2 (Ciriri)*	**2**	**1**
	*Ihimbi (Kalehe)*	**1**	**1**
	*Kahungu (Katana)*	**2**	**1**
	*Walungu*	**1**	**1**
Ituri province	Location		
	*Kigonze (Bunia)*	**2**	**1**
	*Lita (Bahwere)*	**2**	**1**
	*Rwampara (Shari)*	**2**	**1**
Total		20*	13**

*20 interviews with key informants (6 females, 14 males).

**13 FGDs included 139 participants (77 females, 62 males).

### Findings Related to the Elements of the Health System Dynamics Framework

The dominant recurring themes and subthemes reflecting the health system dynamics framework (and their interactions) were identified and reported (Additional Files 4 and 6).

**Context:** « There’s also insecurity, people had been robbed, killed, slaughtered » [FGD participants - Kalehe].

In Eastern Congo, the provision of basic healthcare services is deeply influenced by broader societal changes, including ongoing political regime evolutions, public sector organization, and public financial management. Study participants characterized the context for organizing basic healthcare services as both complex and challenging:

« There used to be war, and there are war-displaced people in this community » [FGD participants - Kalehe].

“People come from battlefields to live and take refuge here. When they arrive, we are obliged to accommodate them after getting permission from the local authorities.”[FGD participants - Bujovu].

“There are a large number of vulnerable people in this community including the elderly, disabled, widows, and orphans.” [FGD Participant Bujovu].

**Population:** “…members of the community are involved in the changes that affect them”. [Interview participant Katana].

This is about people as producers of health and health care, with particular attention to the activities of individuals and the collective action of groups in the community such as patient organizations; peer groups, and informal caregivers. Study participants perceived the population as producers of health and health care and also as potential actors of change as described in the following quotations from interviews:

“Grassroots involvement can bring about changes in health, for example in attitudes. If someone falls ill, and the grassroots is involved, it's a matter of consulting the nurse or doctor to prescribe the right medicines for the patient. [KII Participant Bukavu].

Despite emphasizing community participation, there were some specific challenges to making community producers of their health as described in the following quotations from interviews:

“I can say that members of the community are involved in the changes that affect them. But things haven't gone very well over the last six months. I say that because involving the community in the decision-making process means that the community can be informed when the price of medicines goes up or down. This can also prevent escapes, as the community will be kept informed of the difficulties faced by the health center.” [KII Participant Katana].

While challenges exist in making communities effective producers of their health, there are also significant opportunities for change, thereby creating more resilient and self-sufficient healthcare systems, as noted by one participant:

“Some actions need to be carried out at the local level, such as road maintenance, water sources, traditional farming, etc. You don't have to wait for a government salary to start a farming project at home.” [FGD Participant Walungu].

**Leadership and governance:** “…the way to remedy all this is to create leadership in the community. The leader can advocate at the highest level. They can set up health facilities in the community, and create jobs for the good of the community. We can also benefit from self-help through leadership initiatives.”[FGD Participant Walungu].

Leadership and governance play crucial roles in ensuring that strategic policy frameworks are in place and that these frameworks are supported by effective oversight, coalition building, appropriate regulations, incentives, attention to system design, and accountability. Participants described how leadership and governance have been perceived by community members in several ways.

**For instance, **community members recognize the importance of having clear policies that guide healthcare provision. In the absence of these, the community takes the initiative:

“People can organize themselves into families to set up a family fund to help the sick in the event of illness. In our extended family, we all meet at the end of each month. During these meetings, we make small contributions, and in the event of a serious case (e.g. surgery for a family member), we draw on the family fund.” [FGD Participant Kadutu].

“Good cooperation between the health center and the community can ensure that people are willing to go to the health center.” [FGD Participant Goma].

“I said that people should be encouraged to join mutual health insurance schemes.” [FGD Participant Goma].

They seem to organize themselves, for instance, in Rotating Credit and Saving Associations (ROSCAs), and try to link up with diverse supportive arrangements, such as micro-health insurance, to ensure that healthcare providers and facilities receive payment.

**Organization of resources:** “The majority of the population has very limited resources. “[KII Participant Kirotche].

This is about finances (acquisition and allocation of financial resources in such a way that it effectively contributes to attaining the desired goals and outcomes); human resources (health workers are available, competent, and performing up to standard); infrastructure and supplies (assuring that there are enough health facilities, which are equipped, maintained, and adapted to the specific needs of those making use of it); knowledge and information (being shared between people at different levels). Study participants highlighted several critical barriers to effective healthcare service delivery, emphasizing the significant impact of resource limitations. The following quotations from the interview data illustrate the main challenges identified by participants:

“…if someone falls ill and you take them to hospital, if they don't pay a deposit, we won't take them in.” [FGD Participant Kadutu].

“We've found that patients don't come to the health center because they're afraid of the bill for treatment. They don't have the means to pay the bill.” [KII Participant Masisi].

“Sometimes we don't like the way some of the nurses behave, some focus on those who have money, others focus much more on their tribe and ignore the others who are not theirs.” [FGD Participant Rwampara].

“The healthcare staff have to be welcoming; they have to be ready to receive patients. You arrive at the health center, they give you a warm welcome, they treat you and you go home feeling at ease. The way they welcome you can ensure that the next time you go back to the health center. But if you've been neglected, you won't be able to come back the next time.” [FGD Participant Kirotche].

“People may need to go to the health center for treatment, but given the state of the road, people are obliged to send someone to buy them medicines so that they can start self-medicating at home.” [FGD Participant Walungu].

“The second problem was the lack of health structures; medical structures. When I was young, there was only one health center. The Mabingu health center didn't exist, etc. There were insufficient health structures. The health center that did exist didn't have enough medical staff either; there were only two of them. People come from all parts of the community to access care at this health facility. Now we're having problems with capacity.” [FGD Participant Katana].

“Even if the revenue is small, the drugs are still missing. Something has to be done about the supply chain. The health center needs to be supplied with medicines regularly. I still haven't understood; here, the medicines are in short supply when the number of patients is small. But when it's high, we have more medicines. [FGD Participant Kirotche].

“After treatment, people have difficulty paying 20,000fc to the health center. We may also want to fight against health inequalities, but sometimes the medicines at the health center are insufficient. “[KII Participant Kirotche].

“Most patients leave the health center because there has been no change since they were admitted, due to the lack of appropriate medicines.” [FGD Participant Bahwere].

While many challenges exist, some study participants identified key enablers of effective healthcare service delivery. These enablers highlight the importance of infrastructure, distance, geographic location, and transportation. The following quotations from the interview data illustrate these enablers:

“In this community, we already have good health care because of the multitude of health centers. Before, you might have had to travel 4km to get treatment. But now, if you fall ill, there are at least 4 health centers within 500km. This gives people easy access to care. Health centers have become numerous.” [FGD Participant Kadutu].

“Pregnant mothers are also referred by putting them on motorbikes. ” [FGD Participant Masisi].

“The people who are near the health center are easily made aware but those who are a bit further away are not made aware.” [FGD Participant Masisi].

#### Service Delivery

This refers to the process through which providers, health facilities, programs, and policies are coordinated and implemented to reach the goals of the health system.

Study participants classified service delivery in several ways, focusing on the individuals, families, or the total population aspects; the need for permanent availability versus intermittent scheduling; and the extent to which services are transaction-intensive, discretionary, and subject to information asymmetry.

Study participants distinguished between organized and traditional basic healthcare services delivery as illustrated in the following quote:

“Poverty pushes some members of the community to go to traditional healers and temperance doctors.” [KII Participant Masisi].

Study participants also distinguished public from private basic healthcare services delivery as described below:

“So trust contributes a lot more to changing behaviour. As a result, people have started to lose confidence in these state structures and agree to pay money to go to private facilities.” [KII Participant Bujovu].

“There are churches where, when the sick person is brought in, the pastor takes care of him, and if God speaks to him, he can show him that the illness requires a great deal of prayer or the intervention of nursing staff.” [FGD Participant Kadutu].

#### Values and Principles

Values and principles significantly influence the health system, especially through the power structures and relations within society. These values affect processes such as effectiveness, efficiency, and sustainability. At the community level, a major challenge is finding a balance between the content of a basic healthcare service package and the perceived needs of the vulnerable population. This challenge is exemplified by the following quotation from the interview data, which highlights the unbalanced right of access to basic healthcare:

“So in our community, we don’t even have check-ups to find out how my state of health is, which is the opposite of the situation in other countries where it’s systematic to have check-ups or practically complete examinations that give an idea of a person’s state of health.” [KII Participant Kadutu].

Despite the challenges, participants identified several potential enablers that could improve access to basic healthcare services:

“To facilitate community access to medical care at the health center, the health center should have partners who will assist patients by making medicines available at the health facility and health workers must be well paid. [FGD Participant Katana].

“We need to have a health mutual that can help even the most vulnerable people to access health care, regardless of their meager means, because the community, being united, will make it easier for the whole population to access health care.”[FGD Participant Rwampara].

#### Outcomes and Goals

While the outcomes of a health system include *access* and *coverage,* which are important determinants in the utilization and actualization of health services; the goals are the expected impact in terms of improved health and social and financial protection, and responsiveness entails reacting effectively to the needs and demands of the population and its different subpopulations and vulnerable groups. The following quotation from the interview data exemplifies the major challenges faced by both healthcare providers as well as the most vulnerable users in the Eastern Congo:

“The DRC is a poor country with mixed health results. In addition, there are serious inequalities in health in the DRC according to social rank, with most of the poor having very low levels of access to healthcare in society and often suffering marginalization, discrimination, and abuse.” [FGD Participant Bahwere].

Another community member highlighted how the unfavorable geographic location of their community adversely affects their access to healthcare and coverage:

“But there are areas where access to the hospital is not easy because of the long distances involved, or there simply aren't any roads. Some people have to travel 10 km to get to hospital.“[KII Participant Bukavu].

## Discussion

This qualitative study utilizes the health system dynamics framework^42^ to identify what factors contribute to inequality n the provision of basic healthcare in the Eastern Congo as perceived by community members in a post-conflict area. During prolonged crises, health service analysts often hesitate to undertake comprehensive studies, opting instead for narrower, more manageable topics. However, in such contexts, understanding the dynamics requires a coherent recovery framework is essential for guiding stakeholders through the challenging transition from conflict to peace.^43^ By utilizing a health system dynamics framework in this study, we aimed to enhance our understanding of health system strengthening processes that comprehensively consider various dimensions of the health sector in an interconnected manner. The thematic analysis revealed a wide range of factors (situated on different levels of the HSDF) contributing to inequality in the provision of basic healthcare in the Eastern Congo as perceived by community members.

Guided by the HSDF, the study objectives, and the research questions, 10 major themes were identified as follows: context; population; leadership and governance; organization of resources; service delivery; values and principles; and goals and outcomes.

Our findings reveal that organizing basic healthcare service delivery in the Eastern Congo is complex and challenging due to several intertwined factors. While most of the contributing factors were perceived as barriers (having a negative influence on basic healthcare service delivery), some were perceived as facilitators (having a positive influence on basic healthcare service delivery) (Additional File 5). Our findings are consistent with several studies about service delivery to the most vulnerable populations in fragile environments.^15,44–47^ These results are important as they show cross-thematic linkages, suggesting that addressing issues related to healthcare service delivery in one theme could potentially have a positive impact effect on the other(s).

Study participants mainly refer to their vulnerability within a changing health-related context (including resource mobilisation, health centre management rationalisation, and dynamic community participation). These findings are consistent with other recently published studies, suggesting that the local health system strengthening process must be specificlly adapted to the context.^48,49^ In addition, our study shows that socio-demographics are strongly associated with inequality in the provision of BHS and universal health service coverage. Previous studies in Africa,^50–53^ and Asia^54^ have shown that socio-demographic factors were associated with healthcare service provision.

The study findings highlight the crucial role of the population as active producers of health and potential change agents. This suggests that emphasizing adequate community participation in the decision-making processes is essential for basic health care services delivery. Previous studies have recognized community participation as an essential driver of healthcare service delivery.^55,56^ However, community participation may be troubled by bureaucratic challenges, limited recognition, and engagement despite their apparent contributions.^57^

The study findings highlight the poor strategic policy framework to guide local-level communities in the provision of basic healthcare services, which may lead to fragmented healthcare delivery, inefficiencies, and disparities in the quality and availability of services to the population. For example, the majority of the participants expressed their concerns about a lack of involvement by the local government during resource allocation as a negative contributing factor. Previous studies have shown that good leadership and governance appeared to be associated with better coordination, as they promote responses that are relevant and aligned with the country’s priorities.^58,59^

Some studies examining ROSCA in developing countries suggested beneficial associations of participation in ROSCA with health outcomes.^60,61^ Thus, examples that might promote responses that are relevant and aligned with basic health care services delivery in eastern Congo should include government support for ROSCA’s expansion. ROSCAs are informal financial institutions prevalent in communities with limited access to formal financial services. They involve a fixed group of participants who contribute money regularly to a common pot, which is then allocated using various mechanisms like lotteries or auctions.^62^ An important prerequisite for the effective functioning of ROSCAs is the existence of strong mutual trust among the participants. However, this study shows that this can be an issue in eastern Congo, and should get attention in the course of further research.

For DRC, the government should take responsibility for the ‘stewardship’ of the health system,^41^ which involves ensuring strategic policy frameworks exist and are combined with effective oversight, coalition building, the provision of appropriate regulations and incentives, attention to system design, and accountability.

The study findings identify several critical barriers and facilitators related to effective healthcare service delivery in the Eastern Congo. These factors highlight the complexities and variations in how healthcare services are provided and accessed. Previous studies in post-conflict settings have documented an extreme lack of resources and poor leadership,^63–65^ highlighting challenges to basic healthcare delivery within this context (such as the insufficient financial, material, etc resources to ensure health workers’ regular payment, medications’ availability) and key areas that could benefit from changes in service delivery. For the DRC there is a need to think critically about effective resource allocation within the broad context of health system reform. Opportunities to discuss health resource reallocation in the Eastern Congo are encouraged in future studies.

The findings pertain to the classification of basic health service delivery methods to meet the healthcare needs of the vulnerable population in the Eastern Congo. This classification helps in understanding and organizing different approaches to ensure that healthcare services are effectively reaching those in need. For instance, the limited availability and accessibility of health services were perceived as one of the leading contributing factors to basic healthcare service delivery. Many study participants still considered financial barriers, healthcare care organization, and the quality of care being provided as important challenges to delivering BHS to the population in need. Our findings are generally consistent with previous studies on how limited availability and accessibility of health services negatively influence basic healthcare service delivery. In post-conflict Somalia, for example, inequity in the availability of health services for the poor and those living in rural areas was observed in reduced care to the population in need.^66^

The finding of this study reveals that the healthcare system in the Eastern Congo is pluralistic. This means it consists of multiple overlapping systems and providers, each playing a significant role (influential) in delivering healthcare services. A system cannot function without its values, and its output is not meaningful without meeting system values first. Given that these values and principles (equity, efficiency, solidarity, autonomy, and sustainability) underpin a high-quality health system,^23^ their identification can inform response strategies for the Eastern Congo.

Finally, the study finds that many service providers and potential service users still consider access to basic healthcare services challenging, potentially resulting in reduced coverage. Factors such as violence, poverty, negative perceptions of the healthcare system by potential users and service providers, lack of trust, lack of resources, unfavorable geographic locations, and lack of transportation continue to negatively influence care-seeking choices in the Eastern Congo. For instance, our study found that the provision of BHS is restricted by the distance to functional health facilities coupled with the lack of transportation, suggesting that where there are poor transport systems or lack of, vulnerable populations find it difficult to access the needed healthcare. These findings are in line with earlier overviews.^67–70^ Also, many participants voiced a negative perception of the healthcare system in the Eastern Congo. In this context, populations have endured over two decades of conflict including human rights violations, displacement, loss of economic opportunities, and disruption to health and social services.^71^ Multiple armed groups contribute to violence beyond imagination.^72,73^ In response to this situation, the WHO has advanced a holistic concept of combatting violence at levels ranging from interpersonal to societal.^74^ Proposed mechanisms for how health systems and providers contribute to peace include conflict management, solidarity, strengthening the social fabric, voicing dissent, and restricting the destructiveness of war.^75^

Although contributing factors were distinct, they seemed interrelated and centered around a lack of trust and distrust in the healthcare system, which could result in the community engaging in self-medicating, and becoming hesitant to utilize healthcare services. Our findings are consistent with previous studies highlighting the importance of trust and its influence on people’s decisions about whether they go for healthcare, and where they go for it. The relationship between trust and healthcare delivery in fragile environments is known. For example, a population study in the DRC identified low trust in institutions and beliefs as being associated with a decreased likelihood of adopting preventive behaviors.^76^ Another study found that if people find health services to be unacceptable and are unwilling to use them, they may remain uncovered^1^ even if services are technically in place.^77^ This suggests that community trust in BHS delivery is most likely to be influenced by similar factors.

### Study Strengths and Limitations

This qualitative study specifically explores community perspectives on inequalities in the provision of BHS for the most vulnerable populations in the Eastern Congo. By doing so, we have given voice to the community members in this challenging context - allowing them to become actors of change in the long term.

A key strength of the health system dynamics framework is its ability to facilitate comprehensive interfaces across several crucial dimensions. It enables the examination of population-health system interactions, the organization of service delivery, and the interactions between service delivery and the broader context, all of which significantly impact goals and outcomes. This holistic approach ensures that the complexities and interdependencies within health systems are thoroughly understood and addressed, ultimately promoting more effective and equitable health interventions.

However, there are several limitations to this framework. One major concern is the risk of oversimplification or omission of key elements, as the framework allows for focusing on elements of interest during its application. This can result in a lack of accuracy and a failure to capture all aspects involved in the delivery of basic healthcare services. Another limitation is the challenge of applying such a theoretical framework to real-world healthcare delivery contexts. The abstraction of the framework, combined with the complexity of health strengthening and delivery systems, can make it difficult to effectively support the delivery of basic healthcare services. Additionally, the lack of knowledge and limited understanding of the healthcare system in post-conflict settings can hinder the development of a system that adequately supports the provision of basic healthcare.

Furthermore, the study has followed the consolidated criteria for reporting qualitative research (COREQ),^33^ which is part of the initiatives designed to encourage improvement in the quality of reporting of qualitative studies. However, due to COVID-19 restrictions, and security constraints, interviews and FGDs were limited to the Eastern Congo provinces of North Kivu, South Kivu, and Ituri, which are culturally, politically, geographically, and economically specific, making our findings not generalizable across the DRC. Future studies may want to explore factors that affect the provision of BHS in other provinces of the DRC. Finally, the study setting is characterized by a lack of trust and even distrust among service users and providers. Hence, responder bias might be expected with participants potentially under or over-reporting contributing factors to healthcare delivery in this environment. In an attempt to mitigate this, the researcher took great care to observe the way focus group participants interacted during the group interview. The researcher was sufficiently involved in the group discussion to fulfill the role of facilitator, but not so dominant as to bias or inhibit discussion.

### Implications for Policy and Practice

This study identified a range of factors that impact the provision of basic health care services in the Eastern Congo. Understanding these factors is important for helping community members, healthcare providers, and policymakers to design a better, sustainable healthcare system able to provide universal basic health to the most vulnerable groups. To better serve the communities in the Eastern Congo, there is a need for supportive leadership, effective resource allocation, and rebuilding trust between healthcare providers and users. In addition, there is a need to challenge policies that do not address these factors, as ensuring the efficacy of public services for the most vulnerable populations is a public responsibility. As such, the state plays a crucial role in establishing the policy framework that regulates and monitors basic service provision.^78^ This suggests that ensuring the correct role of the state should be an integrated component of any service delivery strategy.

## Conclusion

In conclusion, this study explored the factors contributing to inequality in the provision of BHS to the most vulnerable populations in the Eastern Congo as perceived by community members, including healthcare service providers and potential users. The following factors were perceived as key areas enabling or hindering healthcare provision to the community: (1) the context for organizing basic healthcare service delivery is complex and challenging; (2) the population plays a crucial role as an active producer of health and potential change agents; (3) there is a poor strategic policy framework to guide local-level communities in the provision of basic healthcare services; (4) Several critical barriers and facilitators related to effective healthcare service delivery were identified; (5) the classification of basic health service delivery methods to meet the healthcare needs of the vulnerable population; (6) the healthcare system is pluralistic and consists of multiple overlapping systems and providers; and (7) Many service providers and potential service users still consider access to basic healthcare services challenging, potentially resulting in reduced coverage. This suggests that if BHS is to be provided to the most vulnerable populations in the Eastern Congo, important changes in these contributing factors are required. Thus, there is a need to critically rethink a healthcare delivery system that addresses these contributing factors in the context of the Eastern Congo. To be successful, such a system should be contextually relevant to the community, and it should involve all stakeholders in the planning, implementation, and ongoing monitoring.

Elsewhere, studies reveal that strengthening public sector health systems to increase the global quality of life may take years. However, it must be integrated, equity-oriented, built on primary healthcare initiatives, and transparent to be achievable. The Rwandan health system development and strengthening initiatives provide evidence that these initiatives can be replicated, while also highlighting the significant concern of implementing these supporting efforts and scaling them from the local to the national level.^49^

Similarly, Uganda has achieved success in scaling up access to ART and reducing the number of children newly infected with HIV. Approaches such as benchmark tools, which have been applied to assess health system readiness to scale up newborn survival interventions, could be applied to national HIV/AIDS interventions with specific actions and lines of accountability for addressing unmet benchmarks before scale-up and ongoing monitoring of health system performance.^79^

Finally, the experience from Somalia shows that expanding health services or developing new initiatives will require strong partnerships between the public and private sectors. Simple, practical, and straightforward joint plans, which are relevant and effective in addressing the country’s health needs, are a better option. These plans are less donor-dependent, more financially sustainable, and easier to transition control once development partners leave the country or their support diminishes.^66^

Overall, although the impact of conflict on the health system can be felt for years after the state has entered the post-conflict phase, it also provides an opportunity for reforms of the affected state’s health sector.^80–86^

## Supplemental Material

Supplemental Material - Community Perspectives on Inequalities in the Provision of Basic Healthcare Services for the Most Vulnerable Populations in the Eastern Congo: A Qualitative Study Supplemental Material for Community Perspectives on Inequalities in the Provision of Basic Healthcare Services for the Most Vulnerable Populations in the Eastern Congo: A Qualitative Study by Dieudonné Bwirire, Rik Crutzen, Rianne Letschert, Edmond Ntabe Namegabe, Bonfils Cheruga, Juliette Mukwege, Trésor Amisi Kasaya and Nanne de Vries in Community Health Equity Research & Policy.

## Note

1. In this context, uncovered refer to “acceptability coverage”, which means people who are willing to use the service but do not because of the health system distrust. As a result, they may not do well according to the index of essential health service coverage indicators that were selected by the UN as SDG indicator 3.8.1

## Appendix

### Abbreviations

AIDS: Acquired immunodeficiency syndrome
ART: Antiretroviral therapy
BHS: Basic healthcare services
DRC: Democratic Republic of the Congo
ECHO: European Civil Protection and Humanitarian Aid Operations
FCAS: Fragile and Conflict-Affected Settings
FGD: Focus group discussions
HIV: Human immunodeficiency virus
HSDF: Health System Dynamics Framework
HSSS: Health system strengthening strategy
IDP: Internally Displaced People
KII: Key individual interviews
RECO: Relais Communautaire (community liaison officers)
RISD: Research Initiatives for Social Development
ROSCAs: Rotating Credit and Saving Associations
SDG: Sustainable Development Goals
UHC: Universal health coverage
UHC SCI: Universal health coverage service coverage index
UNDP: United Nations Development Programme
UN: United Nations
WHO: World Health Organisation
