# Supplementary material for: Community Perspectives on Inequalities in the Provision of Basic Healthcare Services for the Most Vulnerable Populations in the Eastern Congo: A Qualitative Study
Source: Community Health Equity Res Policy. 2025 Feb 17;46(2):195–208. doi: 10.1177/2752535X251321286 (PMC12627245; doi:10.1177/2752535X251321286)
Supplement: Supplemental Material - Community Perspectives on Inequalities in the Provision of Basic Healthcare Services for the Most Vulnerable Populations in the Eastern Congo: A Qualitative Study [file sj-pdf-1-qch-10.1177_2752535X251321286.pdf]

Additional file 1 of community perspectives on inequalities in the provision of basic healthcare services for the most vulnerable populations in the Eastern Congo: A Qualitative Study.

Consolidated criteria for reporting qualitative studies (COREQ): a 32-item checklist

Developed from: Tong A, Sainsbury P, Craig J. Consolidated criteria for reporting qualitative research (COREQ): a 32-item checklist for interviews and focus groups. *International Journal for Quality in Health Care*. 2007. Volume 19, Number 6: pp. 349 – 357

No. Item	Guide questions/description	Reported on Page #
Domain 1: Research team and reflexivity		
Personal Characteristics		
1. Interviewer/facilitator	Which author/s conducted the interview or focus group?	All interviews and focus group discussions were conducted by a trained local researcher (BC), who had many years of experience with the Congolese healthcare system. In addition, two assistant field researchers provided support. They all had training and experience in qualitative data collection and qualitative research methodologies (Pages 5 & 6)

2. Credentials	What were the researcher’s credentials? E.g. PhD, MD	The main local researcher is a medical doctor with a master's degree in public health. The two field assistants are both engineers-agriculture.
3. Occupation	What was their occupation at the time of the study?	Consultants’ data collection at the<br><br>Research Initiatives for Social Development (RISD), in the Democratic Republic of the Congo
4. Gender	Was the researcher male or female?	All interviews and group discussions were conducted by the main local researcher, who is a man. He was assisted by one female (JM) and one male (TAK) (Page 6).
5. Experience and training	What experience or training did the researcher have?	The interviewer had both training (at Master level) and experience in qualitative research methodology.
Relationship with participants		No
6. Relationship established	Was a relationship established prior to study commencement?	Not reported on page<br><br>Yes

7. Participant knowledge of the interviewer	What did the participants know about the researcher? e.g. personal goals, reasons for doing the research	All participants were briefed on the purpose of the study and understood it. They provided written informed consent using the forms that were approved by the ethics committees. Participants reviewed the participant information documentation before giving their consent to be involved (Page 8).
8. Interviewer characteristics	What characteristics were reported about the inter viewer/facilitator? e.g. Bias, assumptions, reasons and interests in the research topic	All interviews and FGD were digitally recorded using a voice recorder and transcribed verbatim by trained local research assistants using Microsoft Word. Transcripts were finally reviewed for completeness and accuracy against interviews.<br><br>Field notes were taken by JM and TAK during each FGD to capture non-verbal communication (Page 6)

Domain 2: study design		
Theoretical framework		
9. Methodological	What methodological	Thematic analysis was used (Page

orientation and Theory	orientation was stated to underpin the study? e.g. grounded theory, discourse analysis, ethnography, phenomenology, content analysis	6)
Participant selection		
10. Sampling	How were participants selected? e.g. purposive, convenience, consecutive, snowball	The study participants were community members, living in eastern Congo, who had experienced at least one episode of conflict in the last 10 years. A heterogeneous sample of community members was recruited, and study participants were selected purposively. Participants were recruited until data saturation. A snowball sampling was also used to identify participants from the group discussion, this was continued until the field researchers team felt that saturation had been reached. (Page 5)
11. Method of approach	How were participants approached? e.g. face-to-face,	Participants were selected purposively from the local

	telephone, mail, email	communities (Page 5)
12. Sample size	How many participants were in the study?	Twenty individual interviews were conducted with community members, and thirteen FGDs were conducted with a total of 139 participants (67 male, 72 female) (Page 5).
13. Non-participation	How many people refused to participate or dropped out? Reasons?	No participants dropped out.
Setting		
14. Setting of data collection	Where was the data collected? e.g. home, clinic, workplace	Meeting room in a public community center (Page 4).
15. Presence of non-participants	Was anyone else present besides the participants and researchers?	No
16. Description of sample	What are the important characteristics of the sample? e.g. demographic data, date	Detailed descriptions of the main socio-demographic attributes of included participants are provided in Table 1 (Pages 8-9)
Data collection		

17. Interview guide	Were questions, prompts, guides provided by the authors? Was it pilot tested?	Interview guides were drafted by the first author, reviewed by all co-authors, and adapted to each respondent's profile.<br><br>The guides were tested and adapted based on feedback received following initial fieldwork (Page 6)
18. Repeat interviews	Were repeat interviews carried out? If yes, how many?	No
19. Audio/visual recording	Did the research use audio or visual recording to collect the data?	All interviews were digitally recorded using a voice recorder and transcribed verbatim by trained local research assistants using Microsoft Word. (Page 6)
20. Field notes	Were field notes made during and/or after the interview or focus group?	Field notes were taken by the research assistant during each FGD to capture non-verbal communication. (Page 6)
21. Duration	What was the duration of the interviews or focus group?	Each interview lasted between 25 minutes to one hour (Page 6)

22. Data saturation	Was data saturation discussed?	Participants were recruited until data saturation (Page 5)
23. Transcripts returned	Were transcripts returned to participants for comment and/or correction?	No
Domain 3: analysis and findings		
Data analysis		
24. Number of data coders	How many data coders coded the data?	All transcripts were coded by at least two individuals (Page 7)
25. Description of the coding tree	Did authors provide a description of the coding tree?	Yes, a description of the coding was provided for the thematic content analysis (Page 6)
26. Derivation of themes	Were themes identified in advance or derived from the data?	Themes were generated from the data (open coding, creating categories, and abstraction) (Page 6)
27. Software	What software, if applicable, was used to manage the data?	All interviews were transcribed verbatim using Microsoft Word, they were analyzed inductively<br><br>using Nvivo 12 (Page 6)

28. Participant checking	Did participants provide feedback on the findings?	Yes
Reporting		
29. Quotations presented	Were participant quotations presented to illustrate the themes/findings? Was each quotation identified? e.g. participant number	Pages 9 to 12- also<br><br>included in the supplementary materials
30. Data and findings consistent	Was there consistency between the data presented and the findings?	Yes
31. Clarity of major themes	Were major themes clearly presented in the findings?	Yes (Page 13)
32. Clarity of minor themes	Is there a description of diverse cases or discussion of minor themes?	Yes (Page 13)

Additional file 2 of community perspectives on inequalities in the provision of basic healthcare services for the most vulnerable populations in the Eastern Congo: A Qualitative Study.

Interview Guide for Key Informant (s)

Date completed (dd/mm/yyyy): ______/_______/2020

1. Pre-Interview

This initial discussion will start with the interviewer briefly explaining the purpose of the study - which is to better understand what is needed to address health inequalities among the most vulnerable populations in the post-conflict Eastern part of the DRC.

This interview is dedicated to the opinions and experiences of one person (having more experience) regarding health inequalities in the community/region.

2. Interview Questions

My research so far has focused on mapping out community assets but also identifying how these could be used to develop an optimal basic healthcare delivery system for the most vulnerable in the Eastern part of the DRC. With you, we would like to move on and talk about your experience and opinion related to health inequality.

Have you ever experienced health inequality in your community? What were the circumstances and what was the outcome?

How important is it for your community to have a strategy that effectively addresses health inequalities? Why?

What has been your experience with power relationships in the community? Give me some examples.

Tell me about the most challenging group you have had to get cooperation from in your community. What did you do, were you successful, and why?

Under what circumstances do you work to gain cooperation from your peers, and when do you do it alone? Give an example and explain Also ask why here.

To what extent are you a believer in the involvement of people in changes that affect them (e.g. health inequality)?

It has been said that one of the best ways to address health inequality is to involve each community member in the decision-making process. Tell me about a time when you contributed to a similar process. What did you find positive and/or negative about this?

Currently, most people believe that change has to start at the bottom (local). To what extent have you found this to be true? Why? Give some examples.

What is your sense of the type of change that you think community involvement can make in addressing health inequalities? What potential, if any, is there to address health inequalities, for example?

What do you believe are some of the most prevalent problems you have in addressing health inequality? Give an example.

How do you identify structural factors that could have the greatest future impact on your strategy to reduce health inequality?

What do you need to do to stay up-to-date on the structural factors, which could affect your strategy to reduce health inequalities?

How do you create solutions to address the various factors contributing to health inequalities to meet the health needs of the most vulnerable populations? What information would you rely on to determine the most effective solution?

Tell me about some strategies that you have seen implemented to reduce health inequality. What did they have in common, how were they different and what about them would be repeatable in another community (anywhere else)?

What are some of the best tips and techniques for coping with challenging health circumstances (e.g. access, availability)?

How comfortable are you in initiating changes within your community that would effectively address health inequality? Give me a couple of examples.

Why is it important to have someone in your group who thinks about addressing health inequality from a different perspective? What have you learned from this person(s) in particular?

3. Finish and close

Thank the interviewee for their time and let them know that the transcript will be shared for them to look over.

Reiterate that any edits or changes can be made and that they can withdraw their contribution at any point.

Additional file 3 of community perspectives on inequalities in the provision of basic healthcare services for the most vulnerable populations in the Eastern Congo: A Qualitative Study.

Topic Guide for Focus Group Discussion

Focus Group Introduction

Welcome

Good morning/afternoon and thanks for agreeing to be part of this focus group. We appreciate your willingness to participate.

My name is_____________________________and assisting me is __________________________

Community name: ____________________________________

District: _____________________________ Date: (DD/MM/YYYY) ____ / ____ / 2020

Community name/description:____

# of male participants: ____

# of female participants:____

Ages represented in the group:

16-25 years☐; 26 – 49 years ☐ ; 50-65 years ☐ , Over 65 years: ☐

Are vulnerable groups present?

(# elderly_________________, # women__________________________, # internally displaced persons________________________, and # of the mentally ill__________ etc.)?

Purpose of Focus Groups

We have been asked by Dieudonné Bwirire to conduct these focus groups. As such, we are meeting with different community members to better understand health inequality. Additionally, we want to know what you would find helpful and assist you in addressing health inequality in your community.

The term health inequality generically refers to differences in the health of individuals or groups. Any measurable aspect of health that varies across individuals or according to socially relevant groupings can be called health inequality.

Insights from this study will help to identify the more optimal design of a health system and the resource constraints for basic health service delivery to the most vulnerable populations in this region.

You were selected because we need your input and we want you to share your honest and open thoughts with us.

Ground Rules

Everyone is asked to actively participate and should be given the space to do so; only one person talks at a time

We would like to listen to everyone’s ideas and opinions

There are no right or wrong answers to questions – just ideas, experiences, and opinions,

which are all valuable

The session will be audio-recorded to help us gather more detailed information about

your responses than the handwritten notes that will be taken by people assisting me, and it will allow us to double-check our data for accuracy (audio recording of this session is dependent on consent from individual participants).

Stay with the group, please don’t have side conversations, and speak clearly to increase recording quality

Turn off or silence cell phones.

Focus Group Questions

Knowledge about health inequality

What does health inequality mean according to you?

What do you know about health inequality in your community?

How does your community experience and assess health inequality?

In your opinion, is there an individual or a group of people who are more vulnerable than others in your community? What are their specific vulnerabilities concerning health inequality? What are their specific health needs?

Do you think there is a person or a group of persons in or outside your community responsible for health inequality? Who? Why?

To what extent is a person or a group of persons aware of current and projected trends affecting their health (status)? Give some examples.

Resource constraints for basic healthcare service delivery

What do you think can happen to an individual or group of people affected by health inequality if they get ill?

How do people understand the link between health inequality and access to basic health? How do they act on it?

If you think someone in your community has been affected by health inequality, what should they do to get assisted?

Probes may include the following:

Traditional healers, traditional birth attendants, pharmacies, health centers, advice from religious and community leaders, advice from the government, advice from community-based organisations, etc.

What are the local community factors that you would consider as facilitating factors/barriers to accessing basic health care? How should these be addressed?

Which resources, capabilities, and skills should a particular group or individuals develop to address health inequality?

How would you allocate resources and planning in anticipation of resource constraints for basic health service delivery? Provide an example.

Design of health service

In your opinion, what community practices/interventions are the most effective in addressing health inequality? Why?

Probes may include the following:

strengths and capacities of different groups, what has worked well in the past for other community issues? How can different groups be involved? Community meetings? Community committees?

In your opinion, what are the characteristics of an optimal health system delivery for basic health service in your community?

Is there anything else you would like to say about under what conditions can the most vulnerable populations get their voices heard when identifying/implementing intervention(s) aiming at reducing health inequality?

Is there anything further you would like to discuss that we did not ask you regarding health inequality that could assist in reducing health inequality?

Thank you so much for coming and sharing your thoughts and opinions with us. That concludes our focus group.

Additional file 4 Overview of the key elements, their meaning, and their features across the three research questions

Elements of the Health System	Meaning	Examples of relevant excerpts and evidence

Dynamics Framework (HSDF)		
Outcomes and Goals	Outcomes are the direct results of the organization of health care delivery. The outcomes of a health system include *access* and *coverage*, which are important determinants in the utilization and actualization of health services. The goals are the expected impact in terms of improved health and social and financial protection, and responsiveness entails reacting effectively to the needs and demands of the population and its different subpopulations and vulnerable groups.	"The DRC is a poor country with mixed health results. In addition, there are serious inequalities in health in the DRC according to social rank, with most of the poor having very low levels of access to healthcare in society and often suffering marginalization, discrimination, and abuse." [FGD Participant Bahwere].<br><br>"…there are areas where access to the hospital is not easy because of the long distances involved, or there simply aren't any roads. Some people have to travel 10 km to get to hospital. "[KII Participant Bukavu].
Values and Principles	The effects of values and principles on the health system are channeled through power structures and relations within the society where certain values relate to processes such as effectiveness, efficiency, and sustainability. An essential function of health system governance is therefore to seek a balance, taking into account the values and principles of actors in the system through a process of negotiation based on fair processes, whilst (1) being accountable to the ultimate beneficiary – the population – and, (2) minimizing harmful effects, especially for the most vulnerable groups.	"We need to have a health mutual that can help even the most vulnerable people to access health care, regardless of their meager means, because the community, being united, will make it easier for the whole population to access health care."<br><br>[FGD Participant Rwampara].<br><br>"To facilitate community access to medical care at the health center, the health center should have partners who will assist patients by making medicines available at the health facility and health workers must be well paid. [FGD Participant Katana].

		"So in our community, we don't even have check-ups to find out how my state of health is, which is the opposite of the situation in other countries where it's systematic to have check-ups or practically complete examinations that give an idea of a person's state of health." [KII Participant Kadutu].
Context	Because health systems are essentially open systems, they are shaped and influenced by wider societal change. This also implies that an analysis of the national context encompasses a governance analysis covering recent evolutions in the domestic political regime; institutional arrangements; the organization of the public sector and public financial management.	"There are a large number of vulnerable people in this community including the elderly, disabled, widows, and orphans. There are also displaced people and refugees." [FGD Participant Bujovu].<br><br>" People come from battlefields to live and take refuge here. When they arrive, we are obliged to accommodate them after getting permission from the local authorities.".[FGD participants - Bujovu].<br><br>« There used to be war, there are war-displaced people in this community » [FGD participants -

		Kalehe].<br><br>« There's also insecurity, people had been robbed, killed, slaughtered « [FGD participants - Kalehe].
Service Delivery	This refers to the process through which providers, health facilities, programs, and policies are coordinated and implemented to reach the goals of the health system. There are several ways to classify the delivery of this wide range of activities and services, including the focus on individuals, families, or the total population; the need for permanent availability or the possibility for intermittent scheduling or the extent to which services are transaction-intensive, discretionary, and subject to information asymmetry.	“We've found that patients don't come to the health center because they're afraid of the bill for treatment. This situation of poverty pushes some members of the community to go to traditional healers and temperance doctors.” [KII Participant Masisi].<br><br>“So trust contributes a lot more to changing behaviour. As a result, people have started to lose confidence in these state structures and agree to pay money to go to private facilities.” [KII Participant Bujovu].<br><br>“There are churches where, when the sick person is brought in, the pastor takes care of him, and if God speaks to him, he can show him that the illness requires a great deal of prayer or the intervention of nursing staff.” [FGD Participant

		Kadutu].
Population	This is about people as producers of health and health care, with attention to the activities of individuals and the collective action of groups in the community such as self-help groups; patient organizations; peer groups, and informal caregivers. It is also about the concept of participation which includes a wide variety of approaches on a scale of increasing empowerment, from mobilizing people to contribute inputs, over common decision-making processes, to increased capacity and autonomously recognizing and acting upon situations. Empowerment at the individual and community levels is widely recognized as an important goal because it contributes to reducing inequities and bringing about desired social change. Empowerment	"Some actions need to be carried out at the local level, such as road maintenance, water sources, traditional farming, etc. You don't have to wait for a government salary to start a farming project at home." [FGD Participant Walungu].<br><br>"I can say that members of the community are involved in the changes that affect them. But things haven't gone very well over the last six months. I say that because involving the community in the decision-making process means that the community can be informed when the price of medicines goes up or down. This can also prevent escapes, as the community will be kept informed of the difficulties faced by the health center. [KII Participant Katana].

	implies the transformation of power relations that is likely to elicit resistance. Empowerment of people, both at the individual and community level, calls for different approaches both at the supply and demand side that improve opportunities for voice, and also in the fair processes of decision-making to ensure that voices are heard and taken into account.	“Grassroots involvement can bring about changes in health, for example in attitudes. If someone falls ill, and the grassroots is involved, it's a matter of consulting the nurse or doctor to prescribe the right medicines for the patient. [KII Participant Bukavu].<br><br>“If you resist treating a pygmy, he'll come with a delegation of pygmies to create misunderstandings at the health center.” [FGD Participant Katana].
Leadership and Governance	This involves ensuring strategic policy frameworks exist and are combined with effective oversight, coalition building, the provision of appropriate regulations and incentives, attention to system design, and accountability. A focus on leadership and governance emphasizes the responsibility of government to ensure that goals are articulated, that necessary systems, capacity, incentives, and information are in place to assure attainment and that all stakeholders can exert appropriate influence on the actions and performance of the system.	“People can organize themselves into families to set up a family fund to help the sick in the event of illness. In our extended family, we all meet at the end of each month. During these meetings, we make small contributions, and in the event of a serious case (e.g. surgery for a family member), we draw on the family fund.” [FGD Participant Kadutu].<br><br>“I think the way to remedy all this is to create leadership in the community. The leader can advocate at the highest level. They can set up health facilities in the community, and create jobs for the good of the community. We can also

		benefit from self-help through leadership initiatives.”<br><br>“Some actions need to be carried out at the local level, such as road maintenance, water sources, traditional farming, etc. You don't have to wait for a government salary to start a farming project at home.”<br><br>[FGD Participant Walungu].<br><br>“I can say that members of the community are involved in the changes that affect them. But things haven't gone very well over the last six months. I say that because involving the community in the decision-making process means that the community can be informed when the price of medicines goes up or down. This can also prevent escapes, as the community will be kept informed of the difficulties faced by the health center. [KII Participant Katana].<br><br>“Good cooperation between the health center and the community can ensure that people are willing to go to the health center.” [FGD Participant Goma].

		“I said that people should be encouraged to join mutual health insurance schemes.” [FGD Participant Goma].
7-10. Organization of Resources	This is about finances; human resources; infrastructure and supplies; knowledge and information.<br><br>*Financing* involves the acquisition, pooling, and allocation of financial resources in such a way that it effectively contributes to attaining the desired goals and outcomes.	“…if someone falls ill and you take them to hospital, if they don't pay a deposit, we won't take them in.” [FGD Participant Kadutu].<br><br>“If you're poor, you don't have anywhere to go to meet your health needs in the event of illness. You don't have the opportunity to go for treatment. “[FGD Participant Katana].<br><br>“We've found that patients don't come to the health center because they're afraid of the bill for treatment. They don't have the means to pay the bill. This situation of poverty pushes some members of the community to go to traditional healers and temperance doctors.” [KII Participant Masisi].

	The health workforce can only meaningfully contribute to the performance of the HS if health workers are available, competent, and performing up to standard. A comprehensive health workforce policy integrates planning and organization of training, recruitment, remuneration, and deployment, adjusted to the evolving models of health care delivery, workloads, and the evolution of the workforce.<br><br>*Infrastructure and supply of pharmaceuticals, technologies, and goods*<br><br>Developing the infrastructure of a health system means assuring that there are enough health facilities within proper reach of the population, which are equipped, maintained, and adapted to the specific needs of those making use of it.	"The majority of the population has very limited resources. After treatment, people have difficulty paying 20,000fc to the health center. "[KII Participant Kirotche].<br><br>"Sometimes we don't like the way some of the nurses behave, some focus on those who have money, others focus much more on their tribe and ignore the others who are not theirs." [FGD Participant Rwampara].<br><br>"The healthcare staff have to be welcoming; they have to be ready to receive patients. You arrive at the health center, they give you a warm welcome, they treat you and you go home feeling at ease. The way they welcome you can ensure that the next time you go back to the health center. But if you've been neglected, you won't be able to come back the next time. [FGD Participant Kirotche].<br><br>"In this community, we already have good health care because of the multitude of health centers. Before, you might have had to travel 4km to get treatment. But now, if you fall ill, there are at least 4 health centers within 500km. This gives

	Essential medicines are a crucial commodity in any health system.<br><br>Information & knowledge Knowledge and understanding are supposed to inform decisions and actions. For this to be effective, knowledge and understanding must be shared in all directions, between people at different levels and similar levels.	people easy access to care. Health centers have become numerous."<br><br>[FGD Participant Kadutu].<br><br>"People may need to go to the health center for treatment, but given the state of the road, people are obliged to send someone to buy them medicines so that they can start self-medicating at home." [FGD Participant Walungu].<br><br>"But there are areas where access to the hospital is not easy because of the long distances involved, or there simply aren't any roads. Some people have to travel 10 km to get to hospital. "[KII Participant Bukavu].<br><br>"The second problem was the lack of health structures; medical structures. When I was young, there was only one health center. The Mabingu health center didn't exist, etc. There were insufficient health structures. The health center that did exist didn't have enough medical staff either; there were only two of them. People come from all parts of the community to access care at this health facility. Now we're having problems with capacity." [FGD Participant

		Katana].<br><br>“The problem is finding the transport costs to Masisi”.<br><br>“Pregnant mothers are also referred by putting them on motorbikes”.<br><br>“The people who are near the health center are easily made aware but those who are a bit further away are not made aware.” [FGD Participant Masisi].<br><br>“Even if the revenue is small, the drugs are still missing. Something has to be done about the supply chain. The health center needs to be supplied with medicines regularly. I still haven't understood; here, the medicines are in short supply when the number of patients is small. But when it's high, we have more medicines. [FGD Participant Kirotche].<br><br>“After treatment, people have difficulty paying

		20,000fc to the health center. We may also want to fight against health inequalities, but sometimes the medicines at the health center are insufficient. "[KII Participant Kirotche].<br><br>"Most patients leave the health center because there has been no change since they were admitted, due to the lack of appropriate medicines." [FGD Participant Bahwere].

Source: Adopted the Health System Dynamics framework from Van Olmen et al. to present the findings of interviews, focus group discussions, and field notes

Additional file 5 Summary of Modifiable and Non-modifiable factors influencing the provision of basic health care services in the Eastern Congo

Key factors	Modifiable factors	Non-modifiable factors
1) Goals and outcomes	Organization of health care delivery.<br><br>Better Health<br><br>Social and financial protection<br><br>Responsiveness<br><br>Users /potential users' satisfaction	Some socio-demographics of people in the community (e.g. being a girl mother, belonging to a specific ethnic/religious group, etc)
2) Values and principles	Overarching values and principles such as effectiveness, efficiency, quality, and	Context of i*nsecurity* in p*rotracted* crises

	sustainability.<br><br>Access, Coverage and Equity<br><br>Safety	
3) Service delivery	Availability of<br><br>functional health centers<br><br>private and public services<br><br>professional and traditional service providers<br><br>Engagement of community liaison officers	Discrimination by health workers<br><br>Lack of incentives to attract health workers
4) Population	Existence of community organizations (e.g.self-help groups; patient organizations; peer groups, and informal caregivers).<br><br>Community participation (increasing empowerment, decision-making processes, etc..)	Unwillingness of community members to join these organizations.
5) Context	Analysis of the national context, including:<br><br>recent evolutions of the political regime;<br><br>institutional arrangements;<br><br>organization of the public sector and public	Absence of historical information on such an analysis of the national context.

	financial management.	
6) Leadership & Governance	Stewardship from state-level healthcare provision programme<br><br>Performance review and coordination	Weak bureaucratic accountability from the local health system.<br><br>Low government attention to healthcare organisation program
7) Finances	External funding for basic health care organisation from donors	Absence of funding (allocation) to basic healthcare organisation local governments' budget.
8) Human resources	Training and supervision of basic health care service providers.	Availability and involvement of untrained traditional service providers.<br><br>Health workers are owed several months of salaries.
9) Infrastructure and supplies	Use of a dedicated/qualified agency for drug procurement and distribution.	Shortage of infrastructure and medical supplies.<br><br>Mobility challenges and access constraints limit the capacity to send necessary supplies and materials to eastern Congo.<br><br>Delays in the delivery lead to increased risk of stock-out of drugs, and other medical materials.
10) Knowledge and	Availability of reporting tools	Lack of reporting tools

information	Adaptation of tools to basic health care provision strategies.	Lack of information among service providers and potential users concerning changes in the tools.

Additional file 6 of Key Elements of the Health System Dynamics Framework and their supporting Subthemes

Key Elements	Supporting Subthemes
1. Outcomes and Goals	Universal access and coverage
	Quality of care
	Responsiveness
	Improved Health
	Social and financial protection
2. Values and Principles	Effectiveness
	Efficiency
	Sustainability
	Solidarity
	Equity

	Autonomy
3. Context	New technologies
	Expectations of patients and service providers
	Availability of information
	Roles of the state in the health and societal sectors
	Domestic political regime
	Institutional arrangements
	Organization of the public sector
	Public financial management
4. Service Delivery	Focus on individuals, families, or the total population
	Permanent availability or intermittent scheduling
	Transaction-intensive - discretionary and subject to information asymmetry
	Private versus public service providers
	Profit versus non-profit service providers
	Formal versus informal service providers
	Professional versus non-professional service providers
	Allopathic versus traditional service providers

	Renumerated versus voluntary
5. Population	Patients or customers
	Funders
	Suppliers of care
	Producers of health and health care
	Individuals
	Collective action of groups in the community
	Participation
	People's empowerment
	Community Voice
	Decision-making
6. Leadership and Governance	Policy guidance
	Coordination of actors
	Regulation of different functions
	Level and actors in the system
	Optimal resource allocation
	Accountability towards the population and all stakeholders

7-10. Organization of Resources	Finance
	Human resources
	Infrastructure and supply of pharmaceuticals, technologies, and goods
	Information Knowledge
